# Reliability and Validity of Assessing User Satisfaction With Web-Based Health Interventions

**DOI:** 10.2196/jmir.5952

**Published:** 2016-08-31

**Authors:** Leif Boß, Dirk Lehr, Dorota Reis, Christiaan Vis, Heleen Riper, Matthias Berking, David Daniel Ebert

**Affiliations:** ^1^ Division of Online Health Training Innovation Incubator Leuphana University of Lueneburg Lueneburg Germany; ^2^ Department of Health Psychology and Applied Biological Psychology Institute of Psychology Leuphana University of Lueneburg Lueneburg Germany; ^3^ Section of Personality, Psychological Assessment, and Psychological Methods Department of Psychology University of Koblenz-Landau Koblenz Germany; ^4^ Section of Clinical Psychology Department of Clinical, Developmental and Neuro Psychology Vrije Universiteit Amsterdam Amsterdam Netherlands; ^5^ EMGO Institute for Health and Care Research VU Medical Centre Amsterdam Netherlands; ^6^ Department of Clinical Psychology and Psychotherapy Friedrich-Alexander-University of Erlangen-Nuremberg Erlangen Germany

**Keywords:** Internet, mental health, evaluation, clinical effectiveness, personal satisfaction

## Abstract

**Background:**

The perspective of users should be taken into account in the evaluation of Web-based health interventions. Assessing the users’ satisfaction with the intervention they receive could enhance the evidence for the intervention effects. Thus, there is a need for valid and reliable measures to assess satisfaction with Web-based health interventions.

**Objective:**

The objective of this study was to analyze the reliability, factorial structure, and construct validity of the Client Satisfaction Questionnaire adapted to Internet-based interventions (CSQ-I).

**Methods:**

The psychometric quality of the CSQ-I was analyzed in user samples from 2 separate randomized controlled trials evaluating Web-based health interventions, one from a depression prevention intervention (sample 1, N=174) and the other from a stress management intervention (sample 2, N=111). At first, the underlying measurement model of the CSQ-I was analyzed to determine the internal consistency. The factorial structure of the scale and the measurement invariance across groups were tested by multigroup confirmatory factor analyses. Additionally, the construct validity of the scale was examined by comparing satisfaction scores with the primary clinical outcome.

**Results:**

Multigroup confirmatory analyses on the scale yielded a one-factorial structure with a good fit (root-mean-square error of approximation =.09, comparative fit index =.96, standardized root-mean-square residual =.05) that showed partial strong invariance across the 2 samples. The scale showed very good reliability, indicated by McDonald omegas of .95 in sample 1 and .93 in sample 2. Significant correlations with change in depressive symptoms (*r*=−.35, *P*<.001) and perceived stress (*r*=−.48, *P*<.001) demonstrated the construct validity of the scale.

**Conclusions:**

The proven internal consistency, factorial structure, and construct validity of the CSQ-I indicate a good overall psychometric quality of the measure to assess the user’s general satisfaction with Web-based interventions for depression and stress management. Multigroup analyses indicate its robustness across different samples. Thus, the CSQ-I seems to be a suitable measure to consider the user’s perspective in the overall evaluation of Web-based health interventions.

## Introduction

### State of Research on Web-Based Health Interventions

In recent years, development and usage of Web-based health interventions have been on the rise, and these interventions show potential in expanding upon established services for preventing and treating impaired health [1]. Many studies so far have shown that Web-based interventions are effective for various conditions, including depression [2,3], anxiety [4,5], sleep disorders [6], alcohol consumption [7], and stress [8-11]. However, there is a lack of published knowledge regarding aspects of effectiveness that are related to external validity, such as the applicability of proven interventions in routine care practice [12]. There are different reasons why evaluators should also examine how affected users directly evaluate the intervention. First, adding to the discussion on the importance of external validity [12,13], the evaluation should go beyond clinical effects that are assessed by health care professionals using observer- or self-rated health measures (eg, assessing depressive symptoms) [14]. The users’ satisfaction with their intervention can be an important source for this metric. We adapted the definition given by Ware and colleagues [15] in our study, which states that satisfaction is a user’s evaluation of the received Web-based intervention. Thus, it provides information beyond what is assessed by health care professionals. Second, it also provides information beyond the design qualities of a Web-based intervention that can be assessed by usability experts [16]. Thus, it delivers important information to service providers so that they can improve their interventions. Third, studying satisfaction can help to successfully implement and disseminate clinically effective Web-based interventions as a part of routine health care [17,18]. Fourth, there are ongoing debates on the relationship between user satisfaction and clinical intervention outcomes [14]. Previous studies found significant correlations between satisfaction with face-to-face interventions and psychological health [19-22] insofar as people with better health were more satisfied. One problem in Web-based interventions is the number of users who do not fully adhere to the intervention protocol [23]. Satisfaction with the delivered intervention may play an important role in understanding adherence to Web-based interventions and vice versa. Some studies, most of which focused on inpatient settings, found that patients who adhere to the intervention are more satisfied than patients who stop participating in the intervention [24]. Investigating the user satisfaction in Web-based health interventions could therefore add to the understanding of such relations. However, there is a strong need for thoroughly studied Web-based measures [25]. To the best of our knowledge, there is yet no validated measure for the assessment of user satisfaction with Web-based interventions.

### Review of Established Satisfaction Measures

Various satisfaction measures have already been developed, such as the Patient Satisfaction Questionnaire Short Form (PSQ-18) [26], the Service Satisfaction Scale-30 (SSS-30) [27], the Satisfaction with Stroke Care Questionnaire (SASC) [28], and the Client Satisfaction Questionnaire (CSQ-8) [29]. Most of these instruments were designed to evaluate health care in hospitals or the general practitioner’s office. In these settings, satisfaction ratings evaluate some dimensions that are not or less relevant to Web-based interventions, including satisfaction with the clinical staff, for example, “I have been treated with kindness and respect by the staff at the hospital,” SASC; the waiting time, for example, “waiting time between asking to be seen and the appointment (date and time) given,” SSS-30; the time spent with a doctor, for example, “Doctors usually spend plenty of time with me,” PSQ-18; or the technical quality, for example, “I think my doctor’s office has everything needed to provide complete care,” PSQ-18. Therefore, satisfaction measures for Web-based interventions must be modified to address their unique characteristics that are not represented in traditional health services. For example, Web-based interventions can be delivered with or without direct contact with health care professionals or can be accessed immediately after registration without any waiting time. In order to address all of these variations, any developed generic measure should be applicable to a wide range of Web-based interventions. Moreover, especially for ease of usage in routine care settings, it is important to have an economically efficient instrument that requires little time to administer by staff and to complete by users. The CSQ seems to be a feasible candidate for adaptation and application to Web-based interventions. The original CSQ has shown good psychometric properties in a study (N=45) to investigate the effects of pretherapy orientation on psychotherapy outcome [30]. The German adaptation has been validated in a sample (N=300) of patients undergoing inpatient treatment within a psychosomatic clinic [31] and has already been integrated as a measure of routine monitoring in inpatient rehabilitation (N=53,177) [32].

The CSQ has also become a widely used instrument for assessing user satisfaction with Web-based health interventions [33-36]. It has been used as secondary outcome in a study comparing Web-based interpersonal psychotherapy and Web-based cognitive behavioral therapy for adults with depressive symptoms, indicating that participants of the first intervention group were more satisfied than the second group [33]. In another study, the CSQ was used as a secondary outcome to compare a Web-based intervention for depression with and without weekly therapist support, indicating that participants of the supported intervention were more satisfied than participants without support [34]. A modified version of the scale was also used in a pilot study of a Web-based screening and brief intervention for student marijuana use, where 95 out of 123 participants (77.2%) were at least moderately satisfied with the intervention [35]. In a previous study by our research group, the CSQ was used as secondary outcome of a Web-based recovery training for employees and indicated that 44 out of 49 participants (89.8%) would recommend the training to a friend in need (item 4 of the CSQ) [36]. However, to our knowledge, there is yet to be a study evaluating the psychometric quality of assessed user satisfaction that tests its factorial structure and its association with indicators of effectiveness such as training adherence and health outcomes in Web-based interventions.

### Aim of the Study

This study aimed to validate an adapted version of the German CSQ to assess the user satisfaction with Web-based health interventions (Client Satisfaction Questionnaire adapted to Internet-based interventions, CSQ-I). First, we examined the internal consistency of the scale, particularly whether the measurement model underlying its 8 items is at least essentially tau equivalent [37], which means that each item measures the same latent variable but with possibly different degrees of precision. Second, considering previous findings [29,31], we expected a single-factorial structure of user satisfaction that would be invariant across both samples in this study. The measurement model and the factorial structure of the scale were cross-validated in 2 independent samples to increase the generalizability of the findings. We further evaluated the validity of the scale by analyzing its correlation with other indicators of effectiveness. The overall evaluation of the psychometric quality of the scale was conducted according to recommendations derived from the COSMIN (Consensus-Based Standards for the Selection of Health Status Measurement Instruments) checklist [38,39].

## Methods

### Study Design

The CSQ-I was evaluated across 2 randomized controlled trials. The first trial was conducted to evaluate the efficacy of a Web-based intervention in preventing the onset of major depressive disorder (Trial Registration: German Clinical Trial Registry DRKS00004709) [40,41]. Participants were recruited from 2013 to 2014 from the general population via newspaper articles, on-air media, and through a campaign of a large insurance company. After completing a Web-based screening questionnaire and telephone interview, individuals aged at least 18 years with elevated depressive symptoms (Center for Epidemiologic Studies Depression Scale, CES-D, ≥16), not having a major depressive episode, were randomly assigned to either the intervention or a control group. The full inclusion and exclusion criteria are described in the efficacy paper of this trial [41]. The intervention consisted of 6 modules that were based on cognitive behavioral therapy. The participants were expected to complete each module within 1 week. All study outcomes were assessed using self-report measures at baseline (T1) and in postintervention assessment after 7 weeks (T2). Study outcomes relevant for the CSQ validation were the reduction of depressive symptoms between T1 and T2, adherence to the intervention, negative side effects, and user satisfaction at T2. The second trial was conducted to evaluate a Web-based stress management intervention in employees (Trial Registration: German Clinical Trial Registry DRKS00004749) [42,43]. Participants of this trial were recruited in 2013 from the general population via newspaper articles, on-air media, and through a campaign of a large insurance company. After completing a Web-based screening questionnaire, individuals aged at least 18 years with elevated symptoms of stress (Perceived Stress Scale, PSS-10, ≥22) were randomly assigned to either the Web-based intervention or a control group. The full inclusion and exclusion criteria are described in the efficacy paper of this trial [43]. The stress management intervention consisted of 7 modules, each to be completed within 1 week. Outcome assessments took place at baseline (T1) and after 7 weeks (T2). The primary outcome was symptom reduction of perceived stress between T1 and T2. Secondary outcomes included adherence to the intervention, negative side effects, and user satisfaction at T2.

### Measures

#### Client Satisfaction Questionnaire Adapted to Internet-Based Interventions

The CSQ-I consists of 8 items measuring global satisfaction with the Web-based intervention (Table 1). On the original German scale [31], respondents rate each of the items on a 4-point Likert-type scale, but wording of response categories differed between the items. For example, the responses for item 1, “How would you rate the quality of service you received?” are rated between 4=“Excellent” and 1=“Poor,” and the responses for item 2, “Did you get the kind of service you wanted?” are rated between 1=“No, definitely not” and 4=“Yes, definitely.” Furthermore, 4 items are scored in inverse to minimize stereotypic response sets. However, after pilot testing (N=15) of the scale, discussion of the results in a focus group consisting of members of our research group, and obtaining advice from experts in the field of Web-based research, we decided to adapt the questionnaire in the following way. First, all questions were rephrased as statements to have constant response scales across the items, ranging from 1= “Does not apply to me” to 4=“Does totally apply to me.” In addition, we replaced the word “service” with “training” in all items because we expected that this wording would be more precise and common for users of Web-based interventions. As in the original version, the scores from all 8 items can be summed to a total score that ranges from 8 to 32. On 5 items (items 1, 2, 3, 5, and 7) participants rate the degree to which the intervention fulfilled their general satisfaction with the quality, kind of training, and amount of help they received. On item 6, respondents rate the degree to which the intervention helped them to deal with their problems. Item 4 assesses the degree to which respondents would recommend the intervention to others. Finally, on item 8, respondents rate their likelihood of using the intervention for themselves again.

#### Clinical Outcome

Depressive symptoms were assessed using the German version of the CES-D [44]. The CES-D is a self-report scale and consists of 20 items (eg, “During the past week I felt sad”), wherein each scored from 0 to 3. The total score ranges from 0 to 60, with a higher score indicating more severe depressive symptoms. A cutoff of 16 is usually regarded as indicating clinically relevant depressive symptom severity.

Symptoms of stress were assessed using the German version of the PSS-10 [45,46]. The PSS-10 assesses the degree to which people perceive their lives as stressful. Participants are asked to answer questions regarding the previous week (eg, “In the past week, how often have you felt that you were unable to control the important things in your life?”) on a 5-point Likert-type scale, with responses ranging from 0 to 4. The total score ranges from 0 to 40, with a higher score indicating higher perceived stress.

#### Adherence

The number of training modules that participants completed was used as the definition of adherence to the intervention in both trials. Full adherence was achieved when participants completed all 6 modules in the depression prevention intervention or all 7 modules in the stress management intervention.

#### Side Effects

The side effects of participating in the training modules were measured with the Inventory for the Assessment of Negative Effects of Psychotherapy (INEP) [47] that was adapted to the training settings. The adapted version consists of 15 items assessing negative changes participants experienced after completing the Web-based training in their social or work environments that they directly attribute to their participation in the training (eg, “During or after finishing the training, I got worse in making important decisions by myself” or “Compared to the time before the training, my relationship to my family is worse”). For the analysis, the negative side effects are counted and summed to a total amount of negative effects. The total score ranges from 0 to 15, with a higher score indicating more negative side effects.

**Table 1:** Item labels and descriptive analysis of the Client Satisfaction Questionnaire adapted to Internet-based interventions.

Item^a^	Sample 1				Sample 2			
	Mean (SD)	n_low_^b^ (%)	n_high_^c^ (%)	L^d^	Mean (SD)	n_low_ (%)	n_high_ (%)	L
1. The training I attended was of high quality or Das Training, an dem ich teilgenommen habe, hatte eine hohe Qualität.	3.48 (0.66)	3 (1.7)	94 (54.0)	0.73	3.45 (0.57)	0	54 (48.6)	0.67
2. I received the kind of training I wanted or Ich habe die Art von Training erhalten, die ich wollte.	3.11 (0.72)	5 (2.9)	54 (31.0)	0.83	3.09 (0.72)	3 (2.7)	31 (27.9)	0.82
3. The training has met my needs or Das Training hat meinen Bedürfnissen entsprochen.	3.13 (0.73)	5 (2.9)	58 (33.3)	0.83	3.05 (0.72)	3 (2.7)	3 (26.1)	0.80
4. I would recommend this training to a friend, if he or she were in need of similar help or Ich würde einem Freund or einer Freundin dieses Training empfehlen, wenn er or sie eine ähnliche Hilfe benötigen würde.	3.36 (0.73)	6 (3.4)	87 (50.0)	0.77	3.50 (0.67)	2 (1.8)	65 (58.6)	0.80
5. I am satisfied with the amount of help I received through the training or Ich bin zufrieden mit dem Ausmaß der Hilfe, die ich durch das Training erhalten habe.	3.18 (0.81)	9 (5.2)	71 (40.8)	0.82	3.12 (0.88)	6 (5.4)	44 (39.6)	0.78
6. The training helped me deal with my problems more effectively or Das Training hat mir dabei geholfen, angemessener mit meinen Problemen umzugehen.	3.25 (0.78)	9 (5.2)	76 (43.7)	0.82	3.09 (0.84)	7 (6.3)	37 (33.3)	0.82
7. In an overall, general sense, I am satisfied with the training or Im Großen und Ganzen bin ich mit dem Training zufrieden.	3.43 (0.80)	8 (4.6)	99 (56.9)	0.90	3.40 (0.70)	1 (0.9)	57 (51.4)	0.89
8. I would come back to such a training if I were to seek help again or Ich würde ein solches Training wieder nutzen, wenn ich Hilfe bräuchte.	3.36 (0.84)	8 (4.6)	101 (58.0)	0.89	3.35 (0.88)	6 (5.4)	63 (56.8)	0.81

^a^ Item scoring: 1=does not apply to me or trifft nicht zu; 2=does rather not apply to me or trifft eher nicht zu; 3=does partly apply to me or trifft teilweise zu; 4=does totally apply to me or trifft voll und ganz zu.

^b^ n_low_=number of participants achieving the lowest possible score.

^c^ n_high_=number of participants achieving the highest possible score.

^d^ L=standardized factor loadings.

### Data Analyses

The analyses were conducted through structural equation modeling using the R package *lavaan* [48]. The covariance matrix was analyzed using the maximum likelihood method with robust (Huber-White) standard errors (MLR), which is asymptotically equivalent to the Yuan-Bentler T2* test statistic [49] recommended for nonnormally distributed data. In the first step, to estimate the internal consistency of the scale, we examined the underlying measurement model of the scale. Essential tau equivalency [37] of the scale indicates that all items can be assumed to assess the same latent variable with the same units of measurement (ie, equal factor loadings). Essential tau equivalency is a necessary assumption for the use of the Cronbach alpha index; if the underlying model violates this assumption, Cronbach alpha will underestimate the reliability of the scale, and McDonald omega should be used instead as a more precise estimate [50]. In the second step, we evaluated whether the one-factor structure proposed by the authors of the original CSQ [30,31] holds across our 2 training samples by conducting multigroup confirmatory factor analyses (CFAs). The idea underlying the estimation of multigroup CFAs is that mean scores of different samples can only be compared in a meaningful way when the requirements of measurement invariance across groups are satisfied. Additionally, multigroup CFAs allow to test if participants in different training groups interpret and respond to the items in the same way. The procedure to establish measurement invariance involves several steps [51,52], which can be described as follows. Configural invariance means that the same common factor structure is shared across groups. Weak invariance indicates that all participants, regardless of their group membership (ie, the received training), respond to the scale items in the same way. Thus, to achieve weak invariance in addition to configural invariance, equivalent factor loadings across the groups are required. Next, we tested for strong measurement invariance by imposing additional constraints on the intercepts of the items (ie, the intercepts of the items were set to be equal across the groups). Strong invariance implies that individuals who have the same score on the latent variable (true score) will obtain the same score on the observed variable regardless of their training group membership.

To assess the fit of the models to the data, we used the following measures: the chi-square statistic, the relative chi-square (χ^2^/ *df*), the comparative fit index (CFI), the root-mean-square error of approximation (RMSEA), and the standardized root-mean-square residual (SRMR). In general, a χ^2^/ *df* value of ≤3.00, a CFI value ≥.95, and RMSEA and SRMR values ≤.08 indicate an acceptable fit to the data. Because the chi-square difference test commonly used to compare nested models is sensitive to sample size, we used additional criteria for model comparisons. For the Akaike information criterion (AIC), models with lower AIC fit the data better than those with higher AIC values. In addition, we used ∆CFI, ∆RMSEA, and ∆SRMR to evaluate test invariance. Considering the number of items and the size of our samples, the following criteria proposed by Chen [53] were applied: ∆CFI<−.005, ∆RMSEA<.010, and ∆SRMR<.005 for testing strong measurement invariance.

To test the convergent validity of the scale, we correlated the CSQ-I scores with the primary clinical outcomes in terms of symptom status at T2 and change of symptoms between T1 and T2. In addition, we compared participants with and without reliable symptom reductions. To assess symptom reductions on an individual level, we examined the number of participants who were classified as having reliably changed according to the reliable change index described by Jacobson and Truax [54]. Participants were defined as having reliably changed if their symptoms declined from T1 to T2 with a reliable change index of greater than 1.96 (8.65 points on the CES-D and 5.16 points on the PSS-10). Furthermore, we conducted explorative subgroup analyses on gender, adherence, and negative side effects from the intervention.

Discriminant validity was evaluated by means of the average variance extracted (AVE). Fornell and Larcker [55] introduced the AVE as an extension of chi-square–based statistics for measuring the goodness of fit between theoretical models with unobservable variables and the empirical data. The index also provides a procedure for establishing discriminant validity. The use of the AVE for this purpose has shown to be robust in various studies, primarily in the field of marketing research. For example, McKinney and colleagues [56] used the AVE as an additional measure of the reliability for the evaluation of a Web-customer satisfaction questionnaire and compared the AVE values with other established measures, such as Cronbach alpha and the composite factor reliability. Liao and colleagues [57] used the AVE as an additional measure to estimate the validity of a planned behavior model, including consumer satisfaction, to predict the customer’s intention toward continued use of Web-based services. In this study, we compared the AVE values for the CSQ-I with the squared correlation estimate between satisfaction and clinical outcome at T2. The AVE was calculated as the total of all squared standardized factor loadings divided by the number of items [58]. Assuming discriminant validity, the AVE should be greater than the squared correlation estimate. This would indicate that user satisfaction is a separate construct, distinguishable from clinical symptoms.

All correlations and subgroup analyses were conducted using IBM SPSS version 22 (SPSS Inc, Chicago, IL, USA). All analyses were done only on complete data samples. Cases with missing values in the outcome variables were excluded from the analyses.

## Results

### Samples’ Characteristics

The depression intervention sample consisted of 201 adults from the German population with clinically relevant levels of depression (CES-D≥16). Complete data on the study outcome variables were available for 174 participants (174/201, 86.6%). The participants were on average 45 years of age (SD 11.84), and 130 were female (130/174, 74.7%; Table 2).

Satisfaction with the Web-based intervention to prevent major depression ranged from mean 3.11 (SD 0.72) on item 2 to mean 3.48 (SD 0.66) on item 1 (Table 1). Each of the items showed a ceiling effect, as at least 54 participants (54/174, 31.0%) achieved the highest possible score (4=“does totally apply to me”) on the items (eg, “I got the kind of training I wanted”). In terms of quality criteria for measurement properties [38], ceiling effects are considered to be present if more than 15% of respondents achieved the highest possible score. The average total CSQ-I score was 26.26 (SD 5.34), with 23 participants (23/174, 13.2%) achieving the highest possible total score and only 1 participant achieving the lowest possible total score. The sample showed a negatively skewed distribution of satisfaction scores (skewness=−1.294, SE=0.184).

The stress management intervention sample consisted of 132 employees from the German population with elevated symptoms of stress (PSS-10 ≥22). Complete data of primary and secondary outcome variables were available for 111 participants (111/132, 84.0%). The participants were on average 42 years of age (SD 9.79). The majority of participants (94/111, 84.7%) were female (Table 2).

The satisfaction ranged from mean 3.05 (SD 0.72) on item 3 to mean 3.50 (SD 0.67) on item 4. At least 29 participants (29/111, 26.1%) achieved the highest possible score on the items (Table 1). The total CSQ-I score was mean 26.05 (SD 4.96) with 14 participants (14/111, 12.6%) achieving the highest possible total score, whereas none of the participants achieved the lowest possible score. The sample showed a distribution that skewed to the left (skewness=−0.909, SE=0.211).

**Table 2:** Samples’ description.

Characteristics		Sample 1 (N=174), n (%)	Sample 2 (N=111), n (%)
**Gender**			
	Female	130 (74.7)	94 (84.7)
	Male 44 (25.3)	16 (14.4)	
	Other^a^	-	1 (0.9)
**Marital status**			
	Single	55 (31.6)	33 (29.7)
	Married or partnership	87 (50.0)	52 (46.8)
	Divorced or separated	32 (18.4)	10 (14.4)
**Education**			
	High school	77 (42.0)	48 (43.3)
	College or university	91 (54.6)	59 (53.1)
	PhD	6 (3.4)	4 (3.6)
**Occupation**			
	Employed full-time	92 (52.9)	85 (76.6)
	Employed part-time	55 (31.6)	25 (22.5)
	Not employed	21 (12.1)	-
	Unemployed	3 (1.7)	-
	Unable to work owing to illness	3 (1.7)	1 (0.9)
**History of psychotherapy**			
	Have not been in psychotherapy	101 (58.0)	72 (64.9)
	Have been in psychotherapy	73 (42.0)	39 (35.1)

^a^Participants who wanted to specify their gender as neither female nor male.

### Test of the CSQ-I Measurement Model

The tau congeneric model indicated that the one-factor model proposed for the original instrument [30,31] was supported in our adapted version. We found that, in both samples, the one-factor model showed an acceptable fit to the data with CFI=.96 and SRMR=.029 in sample 1 and CFI=.95 and SRMR=.035 in sample 2, but the RMSEA values were questionable. In sample 1, RMSEA was .10 (*P*=.002), and in sample 2, RMSEA was .10 (*P*=.02), indicating that the tested model did not perfectly fit the data in sample 2. In the next step, our results revealed that the essentially tau-equivalent model was rejected by the data in both samples. In sample 1, ∆CFI was −.032, ∆RMSEA=.021, and ∆SRMR=.105. In sample 2, ∆CFI was −.052, ∆RMSEA=.031, and ∆SRMR=.140. Also, in both samples, the AICs were lower for the tau congeneric model. Hence, the assumptions for the computation of Cronbach alpha indices were not met [50], and the more precise McDonald omegas were computed instead. Using this metric, the CSQ-I showed very good reliability in both samples, where sample 1 had omega=.95 (bias-corrected and accelerated, BCa, CI .93-.96) and sample 2 had omega=.93 (BCa CI .91-.95).

### CSQ-I Structure Across the 2 Study Samples

In the next step, we examined whether the test scores of the CSQ-I were comparable across the 2 study samples. To do so, we performed multigroup CFAs to test for configural, weak, and strong measurement invariance. The unconstrained model (M1) we used to test for configural invariance fit the data well across sample 1 and sample 2 (Table 3; an extended version of results of this analysis can be found in appendix 1). Furthermore, the model that was used to test for weak invariance (M2) also fit the data well, and the differences in the relevant indices (∆CFI, ∆RMSEA, ∆SRMR) showed that the additional constraints imposed on the data (ie, equal factor loadings) did not significantly alter the fit of the model. Subsequently, we tested for strong measurement invariance by imposing equality on the intercepts (M3). Our results indicated that our data did not support strong measurement invariance, although the corresponding cutoffs were only slightly missed (∆CFI=−0.006, ∆RMSEA=0.001, ∆SRMR=0.005) and the AIC was almost the same: AIC=3581.5 for the model with weak invariance versus AIC=3583.1 for the model with strong invariance. Given that full strong invariance was not supported, we tested for partial strong measurement invariance. The inspection of the means residual matrix revealed a substantial standardized residuum for item 4 (“I would recommend that training to a friend, if he or she were in need of similar help.”) with a value of 4.84. This indicated a lack of invariance for this item across the 2 training samples. When the intercept of item 4 was freely estimated, partial strong invariance was supported (M4).

**Table:** Tests of invariance for the proposed one-factor structure of the Client Satisfaction Questionnaire adapted to Internet-based interventions between sample 1 (N = 174) and sample 2 (N = 111): results of multigroup confirmatory factor analyses with MLR estimator.

Model	χ^2^	*df* ^a^	χ^2^/ *df*	CFI^b^	RMSEA^c^	SRMR^d^
M1 configural invariance	99.2	40	2.5	.964	.102	.031
M2 weak invariance	104.5	47	2.2	.965	.093	.043
M3 strong invariance	120.2	54	2.2	.959	.093	.048
M4 partial strong invariance	111.3	53	2.1	.964	.088	.045

^a^*df*: degrees of freedom.

^b^CFI: comparative fit index.

^c^RMSEA: root-mean-square error of approximation.

^d^SRMR: standardized root-mean-square residual.

### Convergent Validity

In sample 1, the CSQ-I score was significantly correlated with depressive symptoms at T2 (*r*=−.35, *P*<.001; Table 4), indicating that higher satisfaction corresponded to a lower score of depressive symptoms after the intervention. The CSQ-I score was also significantly correlated with a change of depressive symptoms between T1 and T2 (*r*=.27, *P*<.001), meaning that, on average, participants with larger reductions in depressive symptoms appeared to be more satisfied with the intervention compared with those with smaller symptom reductions. The group of participants who met the criteria for reliable reduction of depressive symptoms (102/174, 58.6%) showed significantly greater satisfaction (mean 27.45, SD 4.45) than the group without reliable symptom reduction (mean 24.58, SD 6.03; *t*_172_=3.609, *P*<.001; Cohen’s *d*=0.52).

Most of the participants (134/174, 77.0%) fully adhered to the training protocol by completing all 6 training modules. The participants who fully adhered (n=134, mean 26.48, SD 5.38) did not appear to be more or less satisfied with the training than participants who did not fully adhere (n=40, mean 25.55, SD 5.21; *t*_172_=0.964, *P*=.34). There was no meaningful difference in satisfaction between women (mean 26.17, SD 5.51) and men (mean 26.30, SD 4.87; *t*_172_=0.086, *P*=.93). In this sample, 36 out of 174 participants (21%) reported negative side effects due to the training. In terms of satisfaction scores, participants who reported side effects (mean 26.64, SD 6.26) did not significantly differ from participants without such negative effects (mean 26.17, SD 5.09; *t*_172_=0.472, *P*=.64).

**Table 4:** Means, standard deviations, and intercorrelations of relevant outcomes in sample 1.

Outcome	Mean (SD)	Intercorrelations (*P*)			
		1	2	3	4
1. Satisfaction at T2	26.26 (5.34)	-			
2. Depressive symptoms at T1	26.29 (7.66)	−.04 (.59)	-		
3. Depressive symptoms at T2	17.10 (8.89)	−.35 (<.001)	.35 (<.001)	-	
4. Depressive symptoms T1-T2	−9.19 (9.50)	.27 (<.001)	.49 (<.001)	−.58 (<.001)	-

In sample 2, the CSQ-I score significantly correlated with perceived stress at T2 (*r*=−.48, *P*<.001; Table 5), indicating that higher satisfaction corresponded to a lower score of stress symptoms after the intervention. The CSQ-I score was also significantly correlated with change in perceived stress between T1 and T2 (*r*=.52, *P*<.001), meaning that, on average, participants with larger reductions of perceived stress appeared to be more satisfied with the intervention compared with those with smaller symptom reductions. The group of participants who met the criteria for reliable reduction in perceived stress (60/111, 54.1%) showed significantly greater satisfaction (mean 28.12, SD 3.8) than the group without reliable symptom reduction (mean 23.63, SD 5.10; *t*_109_=5.302, *P*<.001; *d*=1.01). Most of the participants (90/111, 81%) fully adhered to the training protocol by completing all 7 training modules. The participants who fully adhered (n=90, mean 26.81, SD 4.55) were more satisfied with the training compared with the participants who did not fully adhere (n=21, mean 22.81, SD 5.44; *t*_109_=3.492, *P*<.001; *d*=0.85). There was no meaningful difference in satisfaction between women (mean 26.37, SD 4.76) and men (mean 24.13, SD 5.95; *t*_109_=1.681, *P*=.10). In this study, 12 out of 111 participants (11%) reported they experienced at least one negative side effect due to the training. In terms of satisfaction scores, participants who reported side effects (mean 27.75, SD 3.25) did not significantly differ from participants without such negative effects (mean 25.85, SD 5.11; *t*_109_=1.257, *P*=.21).

**Table 5:** Means, standard deviations, and intercorrelations of relevant outcomes in sample 2.

Outcome	Mean (SD)	Intercorrelations (*P*)			
		1	2	3	4
1. Satisfaction at T2	26.05 (4.96)	-			
2. Stress at T1	25.28 (4.60)	.08 (.42)	-		
3. Stress at T2	18.68 (6.27)	−.48 (<.001)	.22 (.02)	-	
4. Stress T1-T2	−6.60 (7.00)	.52 (<.001)	.45 (<.001)	−.74 (<.001)	-

### Discriminant Validity

In sample 1, the AVE values for both measures (CSQ-I AVE=0.681, CES-D AVE=0.446) were greater than the squared correlation between these outcomes (*R*^2^=.123), indicating that the CSQ-I construct explained more of the variance in its items than it shared with the CES-D. In sample 2, the AVE values for both measures (CSQ-I AVE=0.512, PSS-10 AVE=0.411) were also greater than the squared correlation between the CSQ-I and the PSS-10 (*R^2^*=.230), indicating that the adapted CSQ-I explained more of the variance in its items than it shared with the PSS-10.

## Discussion

### Principal Findings

In the evaluation of Web-based health interventions, the user’s perspective should be taken into account [15,24]. For this purpose, Web-based measures with proven psychometric quality are needed [25]. In this study, we investigated the factorial structure, the measurement model, and construct validity of an adapted version of the CSQ in 2 samples of adults who had participated in Web-based health interventions for either preventing major depression or improving stress management.

Multigroup factor analyses on the CSQ-I confirmed the proposed one-factorial structure [29,31] of the original scale across 2 independent samples. Our results showed that, although the assumptions needed for Cronbach alpha were not met, the scale demonstrated excellent reliability through McDonald omega [50] with omega=.95 in sample 1 and omega=.93 in sample 2. These findings correspond to previous studies that showed a very good reliability of the original scale, indicated by Cronbach alpha indices of alpha=.93 [30], alpha=.87 [31], and alpha=.90 [32]. The results on measurement invariance across the groups imply that the factor structure was replicated between the 2 samples; however, this should be interpreted with caution because the differences found in the latent means were due to partial rather than full strong measurement invariance. Although some researchers argue that in order to test for latent means between two samples at least two items must have invariant loadings and intercepts [59], Thompson and Green [60] reason that “in models with equivalent factor loadings but differing intercepts, differences in the means on that measure are a function of both the latent factors and the varying intercepts which can be interpreted in terms of a biased measure” (p149). However, we stress that the differences in the indices comparing weak and strong invariance were very small, indicating that the lack of invariance was marginal.

In line with previous findings [30-32,61], the satisfaction scores were on average very high, indicating that the participants tended to be very satisfied with the delivered intervention. This result may be restricted owing to a ceiling effect [38] because many participants achieved the highest possible satisfaction score in both samples. However, the results showed that participants with reliable symptom reductions due to the received intervention were more satisfied than those without reliable reductions. Thus, these findings indicate the ability of the scale to discriminate between more and less satisfied intervention users, despite potential ceiling effects. Nevertheless, some studies suggest that modifying the response choice pattern from a 4-point format to a 5-point format with three positive choices and two negative choices can increase the variability of satisfaction scores [62-64]. Thus, testing a further adaptation of the CSQ-I response format may be useful in the future. The content validity of the scale was primarily investigated in relation to clinical outcomes in terms of psychopathological symptoms after the intervention and change of symptoms between baseline and postintervention assessment. The correlations between satisfaction and symptoms at the postintervention assessment were *r*=−.35 in sample 1 and *r*=−.48 in sample 2. These results are in line with findings from the original CSQ version with correlation coefficients of *r*=−.34 for satisfaction × psychosomatic symptoms at postintervention assessment [30], *r*=.40 for satisfaction × health condition at discharge [31], and *r*=.40 for satisfaction × health condition at discharge [32]. The correlation between satisfaction and change of symptom severity from baseline to postintervention assessment was *r*=.27 in sample 1 and *r*=.52 in sample 2 in our study. These results also correspond to findings from the original CSQ regarding correlations between satisfaction and psychosomatic symptom reduction of *r*=−.40 [30] and health condition improvement of *r*=.52 [31] and *r*=.60 [32]. In summary, the results of the content validity analysis can lead to the assumption that participants might have rated their satisfaction to be high merely because they felt better after the training. In this case, the satisfaction score would display a proxy measure for the clinical outcome. However, the discriminant validity analyzed in terms of the AVE values of the CSQ-I indicates that the satisfaction measure and the clinical outcome measure assessed different constructs. The relation between satisfaction and adherence remains unclear. In the second sample only, we found a marginal but statistically significant difference between the satisfaction scores of participants who did and did not fully adhere to the intervention. In general, low satisfaction with the intervention is assumed to be associated with low adherence [25]; notwithstanding that individuals experiencing a high burden (eg, due to depressive symptoms) may be under considerable pressure to find relief. Those individuals may also adhere to an existing intervention, although they do not evaluate all aspects of the intervention as favorable. This might rather apply to the participants in the depressive intervention sample than to those in the stress intervention sample.

Nevertheless, some limitations of this study should be taken into account. First, the study dropout rates were very low in both samples (27/201, 13.4% in sample 1, 22/132, 16.0% in sample 2), corresponding to dropout rates in other validation studies [31,61,65]. However, it is possible that participants who did not complete posttreatment assessments may have rated their satisfaction lower than participants who attended the posttreatment assessment [65]. Second, we were not able to control for the interference between satisfaction and postintervention health state in terms of psychopathological symptoms because both variables were assessed at the same time point. Thus, we could not exclude the possibility that the participants’ health state, after participating in the training modules, biased their satisfaction rating. Further experimental studies are needed to investigate the clinical effects of Web-based interventions on satisfaction, using different time points for the assessment of user satisfaction and health outcomes, and also consider follow-up assessments. Third, because we used the same clinical outcome measure for state and for change of psychological health, it was not possible to estimate the predictive impact of both health outcomes on satisfaction independently. Thus, it may be beneficial to use different measures for (1) health condition at the postintervention assessment and (2) change of health over time. Fourth, it was not possible to analyze the impact of adherence on satisfaction. Adherence was operationalized by the number of completed training modules. In both samples most of the participants completed all modules (134/174, 77.0% in sample 1 and 90/111, 81% in sample 2), so that it would not have been of value to determine the correlation between adherence and satisfaction. Future research should use additional measures of adherence (eg, login counts or time spent on the training website) to investigate the construct validity of the scale. Unfortunately, such data were not available for our study. Fifth, the subgroup analyses of gender and negative side effects from the intervention were underpowered. Hence, future studies are needed to explore potential relevant subgroup effects such as gender and negative intervention effects that may influence satisfaction ratings. Finally, one theoretical limitation must be taken into account when using the CSQ-I for the evaluation of Web-based health interventions. There have been previous discussions regarding the usefulness of user satisfaction in assessing quality of health care interventions, mainly because of its construct validity and unclear evidence for its association with other health outcomes [14]. It is important to note that the CSQ-I covers the user’s satisfaction with Web-based health interventions in a broader sense rather than focusing on specific intervention aspects. Thus, it is not clear on which aspects of the intervention the participants actually based their satisfaction rating. Most of the CSQ-I items cover the fulfilled expectancy in terms of the general quality of the intervention, their intention to use it again, or their likeliness of recommending it to other affected people. The items do not cover specific aspects and surrounding conditions of the intervention (eg, usability of the Web-based program, registration and login procedures, psychological and technical guidance) that may also be relevant for clinical success [1] and adherence [16] in Web-based health interventions. Thus, it may be valuable to evaluate additional, more specific quality dimensions of Web-based interventions (eg, technical support, usability, simplicity of the intervention content).

### Conclusions

In this study the CSQ-I has shown to be a robust measure with a clear factorial structure across different samples. Thus, the CSQ-I seems to be a suitable measure to consider the user’s satisfaction in the overall evaluation of Web-based health interventions. It can provide an important source of information for service providers who wish to improve or implement their interventions into routine health care. Furthermore, satisfaction scores derived from the CSQ-I may serve as a useful reference for other people who are seeking help via the Web.

## Appendix

Multimedia Appendix 1

## Abbreviations

AIC: Akaike information criterion
AVE: average variance extracted
BCa: bias-corrected and accelerated
CES-D: Center for Epidemiologic Studies Depression Scale
CFA: confirmatory factor analysis
CFI: comparative fit index
CSQ: Client Satisfaction Questionnaire
CSQ-I: Client Satisfaction Questionnaire adapted to Internet-based interventions
PSQ-18: Patient Satisfaction Questionnaire Short Form
PSS-10: Perceived Stress Scale, 10-item version
RMSEA: root-mean-square error of approximation
SASC: Satisfaction with Stroke Care Questionnaire
SRMR: standardized root-mean-square residual
SSS-30: Service Satisfaction Scale-30
