# Supplementary material for: Reliability and Validity of Assessing User Satisfaction With Web-Based Health Interventions
Source: J Med Internet Res. 2016 Aug 31;18(8):e234. doi: 10.2196/jmir.5952 (PMC5023944; doi:10.2196/jmir.5952)
Supplement: Multimedia Appendix 1 [file jmir_v18i8e234_app1.pdf]

Table 3 (extended) - Tests of invariance for the proposed one-factor structure of the Client Satisfaction Questionnaire adapted to Internet-based interventions between sample 1 (N = 174) and sample 2 (N = 111): results of multigroup confirmatory factor analyses with MLR estimator.

Model	χ^2	*df*[a]	χ^2/df	CFI[b]	RMSEA[c]	SRMR[d]	Comparison	$\Delta\chi^2$ (*df*)	P	ΔCFI	ΔRMSEA	ΔSRMR
M1 configural invariance	99.2	40	2.5	.964	.102	.031						
M2 weak invariance	104.5	47	2.2	.965	.093	.043	M1-M2	4.7 (7)	.70	−.001	.009	.012
M3 strong invariance	120.2	54	2.2	.959	.093	.048	M2-M3	15.7 (7)	.03	−.006	.001	.005
M4 partial strong invariance	111.3	53	2.1	.964	.088	.045	M2-M4	5.9 (6)	.43	−.001	.005	.002

[a]*df*: degrees of freedom.

[b]CFI: comparative fit index.

[c]RMSEA: root-mean-square error of approximation.

[d]SRMR: standardized root-mean-square residual.
